# Association of Skin Cancer With Clinical Depression and Poor Mental Health Days: Cross-Sectional Analysis

**DOI:** 10.2196/80710

**Published:** 2026-01-22

**Authors:** Riona Ray, Mytien Nguyen

**Affiliations:** 1 Marvin Ridge High School, 7117 Stonehaven Drive, Waxhaw, NC, 28173, United States, +1 402-871-2550; 2 Yale School of Medicine, New Haven, CT, United States

**Keywords:** mental health, nonmelanoma skin cancer, depression, sociodemographic variables, analysis

## Abstract

**Background:**

Mental health is becoming increasingly recognized as an important part of overall health, especially for patients with cancer. However, the relationship between nonmelanoma skin cancer and mental health has not been widely studied.

**Objective:**

The aim of this study was to examine the association between nonmelanoma skin cancer diagnosis and 2 key mental health outcomes (ie, clinical depression and the number of poor mental health days).

**Methods:**

This study used the 2023 Behavioral Risk Factor Surveillance System, a nationally representative survey of adults in the United States, which included 312,317 participants. Nonmelanoma skin cancer diagnosis, depression, and self-reported mental health days were analyzed. Logistic regression was used to evaluate the association between nonmelanoma skin cancer and depression, whereas Poisson regression was used to model the number of poor mental health days, adjusting for age, sex, race and ethnicity, education, BMI, income, and major comorbid conditions (other cancers, heart disease, lung disease, and kidney disease).

**Results:**

Individuals with nonmelanoma skin cancer (5086/26,552, 19.15%) reported a lower overall rate of depression compared to those without nonmelanoma skin cancer (61,438/285,765, 21.50%; *P*<.001) but reported more poor mental health days on average (4.54, SD 8.37 d vs 3.20, SD 7.37 d; *P*<.001). After adjustment, nonmelanoma skin cancer diagnosis was not significantly associated with depression (adjusted odds ratio 1.01, 95% CI 0.98‐1.05) and was associated with a slightly lower number of poor mental health days (adjusted rate ratio 0.94, 95% CI 0.91‐0.97).

**Conclusions:**

Adults with nonmelanoma skin cancer experienced a meaningful mental health burden, and unadjusted analyses suggested greater day-to-day distress than among adults without nonmelanoma skin cancer. However, these differences were reduced and no longer significant for depression after adjusting for sociodemographic factors and comorbid chronic illnesses. These findings support the need for mental health screenings and support services in dermatologic and oncologic care.

## Introduction

### Background

In recent years, public health conversations have continued to emphasize the importance of mental health. Mental health is increasingly viewed not as a stand-alone issue, but as a factor that deeply interacts with physical illness, such as cancer [1].

While nonmelanoma skin cancer has obvious physical consequences, it poses serious complications regarding mental health. This aspect has not received sufficient attention in the health care field [2]. Dermatologists and oncologists are facing a dramatic rise in cases of nonmelanoma skin cancer, with melanoma rates doubling over the past two decades [3]. Nonmelanoma skin cancer is one of the most diagnosed malignancies in the world today, with rates for both melanoma and nonmelanoma types on the rise [3]. The relationship between mental state and nonmelanoma skin cancer is a complex feat. While the stress of diagnosis and treatment can create or worsen mental health conditions [4,5], existing mental health conditions can also increase the chances of developing nonmelanoma skin cancer through behavioral, immunological, and systemic mechanisms [6]. Recent evidence shows that approximately 30% of patients with melanoma experience anxiety, and nearly 20% experience depression. The highest risk has been observed among women and young adults [7]. Other studies have confirmed similar trends, showing that psychological distress and the fear of recurrence remain substantial even in patients with early-stage melanoma.

Research conducted recently has started to uncover the complex relationships between mental health and nonmelanoma skin cancer. A 2016 cross-sectional study using Behavioral Risk Factor Surveillance System data found that individuals who had frequent poor mental health days had a significantly higher chance of being diagnosed with the disease of nonmelanoma skin cancer [8].

These data were confirmed even after using the multivariate logistic regression analyses. These analyses suggest a possible link between poor mental health and keratinocyte carcinoma. This could be possible through factors such as dysregulated immune responses [9]. Other studies have shown similar results, mostly highlighting a high dose of psychological distress among patients with cancer [5,8]. Additionally, approximately one-third of patients with melanoma skin cancer require professional mental health care but are not receiving that treatment [1,2].

Further literature reviews on neuroendocrine-immune interactions support the biological plausibility of this connection. Chronic mental distress is a well-known contributor to the disruption of skin immunity, wound healing, and active inflammatory mediators, which can all contribute to the progression of cancer [6]. Additionally, factors including hostility and depression have been connected to melanoma and its treatment outcomes [4]. There is an extremely minimal amount of information regarding the demographic or socioeconomic factors that shape the outcomes of mental health across nonmelanoma skin cancer subtypes [3,10].

This study aimed to address these gaps in knowledge by analyzing the association between mental health disorders and the rate of nonmelanoma skin cancer diagnosis by using the information provided by the Behavioral Risk Factor Surveillance System (BRFSS). Focusing on nonmelanoma skin cancers, assessing the link between nonmelanoma skin cancer and mental health status by sociodemographic factors, such as age, sex, race, income, BMI, and education, will provide critical insights into how mental health influences the risk and experience of nonmelanoma skin cancer.

### Literature Review

Recent studies document consistent associations between multiple indicators of psychological distress and nonmelanoma skin cancer. A proportional meta-analysis of patients with melanoma reported prevalence estimates of 30% for anxiety and 20% for depression, with higher odds observed among women, younger adults, and individuals with lower education levels [7]. Similar findings have been reported in earlier clinical and observational studies, showing elevated levels of psychological symptoms across different stages of melanoma, including treatment and posttreatment phases [5,11].

Beyond symptom prevalence, multiple studies have examined behavioral and biological pathways linking mental health to nonmelanoma skin cancer. Young adults with mental health problems demonstrate higher rates of cancer-related risk behaviors, such as smoking, alcohol use, sleep disturbances, and inactivity, which may contribute to disease development or worse outcomes [10]. Experimental research has also shown that chronic psychological stress alters neuroendocrine and immune signaling, increasing inflammatory activity and harming skin repair processes [6]. Additional studies have reported relationships between melanoma severity and personality traits, such as hostility and depressive tendencies.

More recent research has shifted attention to survivorship and early-stage disease. Patients diagnosed with melanoma have reported reduced emotional well-being. They have also stated persistent uncertainty despite a favorable clinical prognosis. This suggests that psychological effects extend further than cancer itself. Moreover, fear of recurrence has been identified as a primary contributor to ongoing mental distress following the completion of treatment [12]. These findings indicate that mental health challenges in nonmelanoma skin cancer populations can include forms of distress that may not be clinically diagnosed.

Population-based research has identified variation in mental health outcomes among patients with nonmelanoma skin cancer across demographic and socioeconomic subgroups. Studies have shown that mental health service use remains limited, with unmet psychological needs concentrated among older adults and lower-income populations [1,2]. Global assessments have revealed a lower quality of life in regions with lower access to supportive care resources [3].

Globally, the burden of skin disease is high in many regions, especially Asia, and is linked to socioeconomic status and inflammatory conditions [3]. Tools such as the Skin Cancer Index have been developed to measure the quality of life in patients with nonmelanoma skin cancer [13].

## Methods

### Participants

This study used data from the BRFSS, a nationally representative survey conducted by the Centers for Disease Control and Prevention [9]. The data used were from the year 2023. The BRFSS surveys US adults aged 18 years or older, collecting data on health conditions, behaviors, and preventive health practices [9]. This dataset included responses to questions related to nonmelanoma skin cancer, mental health, and sociodemographic characteristics. Participants with missing, refused, or “don’t know” responses were excluded from the analyses to ensure the high quality and reliability of the study.

### Exposure

The independent variable was a self-reported diagnosis of nonmelanoma skin cancer. These individuals did not have to have a current diagnosis; the diagnosis could be from any time in the past. Respondents were asked whether a health professional had ever told them they had skin cancer, including melanoma and nonmelanoma types. Individuals who answered “yes” were categorized as having a nonmelanoma skin cancer diagnosis. Those who answered “no” were the comparison group. Those with missing or ambiguous responses were excluded from the analysis to maintain the integrity of the data.

### Outcomes

The 2 primary mental health–related outcomes that were examined were depression and the number of poor mental health days an individual had. Depression was defined as being diagnosed with a depressive disorder by a health care professional [9,10]. Poor mental health days were based on the number of days during the past 30 days that an individual reported that their mental health was “not good,” including stress, depression, and other emotional issues [1,3]. Respondents with invalid responses were excluded from the analysis.

### Covariates

The sociodemographic variables that were included in the analysis were age (18‐64 and ≥65 years), sex (male or female), race or ethnicity (White only, Black only, Asian only, American Indian or Alaskan Native only, Native Hawaiian or other Pacific Islander only, multiracial, and other), education (did not graduate high school, graduated high school, attended college or technical school, and graduated from college or technical school), and BMI (underweight, normal weight, overweight, and obese). Additional health-related covariates included self-reported diagnoses of other (non–skin) cancer, heart disease, chronic lung disease, and kidney disease. These covariates were specifically selected based on the evidence linking them to mental health and cancer-related outcomes [3,10].

### Statistical Analysis

Descriptive statistics were first used to summarize the distribution of depression status and the number of poor mental health days by nonmelanoma skin cancer diagnosis and sociodemographic variables, including age, sex, race and ethnicity, education level, and BMI. All statistical models were run on the entire BRFSS sample, and individuals without a history of nonmelanoma skin cancer served as the reference group. This allowed a direct comparison between those with and those without nonmelanoma skin cancer. Categorical variables were summarized using frequencies and percentages, while continuous variables were described using means and SDs. Group differences in categorical variables were assessed using Pearson χ^2^ tests, and differences in continuous outcomes were interpreted using independent samples *t* tests. These tests described unadjusted differences between adults with and without a history of nonmelanoma skin cancer.

To examine the association between nonmelanoma skin cancer diagnosis and depression, a multivariable logistic regression model was used [9]. Depression was treated as a yes or no outcome, and a nonmelanoma skin cancer diagnosis (yes or no) was the main comparison of interest. All statistical models were run on the full BRFSS sample, and individuals without a history of nonmelanoma skin cancer served as the reference group in all analyses. This approach allowed direct comparison of depression prevalence and poor mental health days between respondents with and without nonmelanoma skin cancer, instead of limited analyses to only the skin cancer subgroup. Additionally, adjusted odds ratios (aORs) and corresponding 95% CIs were reported.

For the continuous outcome of mental health days, a multivariable Poisson regression model with standard errors to account for potential overdispersion was used. The results were expressed as adjusted rate ratios (aRRs) with 95% CIs, which allowed for the calculation of the relative increase or decrease in the expected number of poor mental health days among individuals with nonmelanoma skin cancer compared to those without, after accounting for sociodemographic factors. Both regression models adjusted for age, sex, race and ethnicity, education level, BMI, household income, and comorbid conditions (other cancer, heart disease, lung disease, and kidney disease). These sociodemographic and health-related variables are independently associated with both mental health outcomes and cancer risk in prior studies. Logistic regression was used for the binary depression outcome, whereas a multivariable Poisson regression model was used for the count-based outcome of poor mental health days. Poisson regression was selected because the outcome represents a count of days within a fixed 30-day period and was not normally distributed, making linear regression inappropriate. Standard errors were adjusted to account for overdispersion. The distribution of days with poor mental health was examined. It was discovered that, although the data showed variability, it did not exhibit sufficient overdispersion to warrant switching to an alternative model. Therefore, the Poisson model was the best option.

All statistical tests were 2 sided. Analyses were conducted using JASP, ensuring appropriate complex survey weighting to reflect the nationally representative design of the BRFSS dataset [9].

### Ethical Considerations

This study involved secondary analysis of publicly available, deidentified data from the BRFSS, administered by the US Centers for Disease Control and Prevention. As the dataset contains no identifiable private information, this study did not constitute human subjects research and was therefore exempt from institutional review board review in accordance with US federal regulations. The BRFSS protocol is reviewed and approved annually by the US Centers for Disease Control and Prevention Institutional Review Board, and informed consent is obtained from all participants at the time of data collection.

## Results

### Overview

Among 433,323 participants in the 2023 BRFSS questionnaire, 312,317 (72.07%) had complete demographic and disease information and were included in the analysis (Table 1). Among the analytical cohort, 154,230 (49.38%) were men, and 158,087 (50.62%) were women. Additionally, 253,634 (81.21%) identified as White participants only. The remaining racial and ethnic distribution included 26,936 (8.62%) Asian only, 6551 (2.10%) Black only, 8865 (2.84%) American Indian or Alaska Native only, 2041 (0.65%) Native Hawaiian or Pacific Islander, 5742 (1.84%) multiracial, and 8548 (2.74%) identifying as other race and ethnicity.

**Table 1.** Characteristics of the study cohort [8].

Characteristics	Values
Skin cancer diagnosis, n (%)	
No	285,765 (91.50)
Yes	26,552 (8.50)
Depression, n (%)	
No	245,793 (78.70)
Yes	66,524 (21.30)
Mental health days, mean (SD)	4.42 (8.29)
Race and ethnicity, n (%)	
White only	253,634 (81.21)
Asian only	26,936 (8.62)
Black only	6551 (2.10)
American Indian or Alaskan Native only	8865 (2.84)
Native Hawaiian or other Pacific Islander only	2041 (0.65)
Multiracial	5742 (1.84)
Other race only	8548 (2.74)
Sex, n (%)	
Male	154,230 (49.38)
Female	158,087 (50.62)
Age (y), n (%)	
18-64	198,394 (63.52)
≥65	113,923 (36.48)
BMI, n (%)	
Underweight	4802 (1.54)
Normal weight	89,431 (28.63)
Overweight	111,680 (35.76)
Obese	106,404 (34.07)
Education, n (%)	
Did not graduate high school	14,184 (4.54)
Graduated high school	73,285 (23.46)
Attended college or technical school	83,761 (26.82)
Graduated from college or technical	141,087 (45.17)
Other cancer, n (%)	
No	275,645 (88.26)
Yes	36,672 (11.74)
Heart disease, n (%)	
No	277,856 (88.97)
Yes	34,461 (11.03)
Lung disease, n (%)	
No	251,263 (80.45)
Yes	61,054 (19.55)
Kidney disease, n (%)	
No	297,584 (95.28)
Yes	14,733 (4.72)

Most of the 312,317 respondents were aged between 18 and 64 years (n=198,394, 63.52%), with 113,923 (36.48%) aged 65 years or older. BMI classifications showed that 4802 (1.54%) were underweight, 89,431 (28.63%) had a normal BMI, 111,680 (35.76%) were overweight, and 106,404 (34.07%) were obese. Educational attainment also varied, with 14,184 (4.54%) not graduating from high school, 73,285 (23.46%) graduating from high school, 83,761 (26.82%) attending some college or technical school, and 141,087 (45.17%) graduating from a college or technical program.

Most respondents did not report a nonmelanoma skin cancer diagnosis, with 285,765 (91.50%) of 312,317 indicating no history of nonmelanoma skin cancer and 26,552 (8.50%) reporting a diagnosis. Additionally, 245,793 (78.70%) participants did not report depression, whereas 66,524 (21.30%) reported having been diagnosed with depression by a health care professional. The high average number of mental health days was consistent with high fluctuations in mental health experiences across many individuals.

Comorbid health conditions were also reported. A total of 36,672 (11.74%) participants reported another form of cancer, 34,461 (11.03%) reported heart disease, 61,054 (19.55%) reported lung disease, and 14,733 (4.72%) reported kidney disease.

The average number of poor mental health days in the past 30 days was 4.42 (SD 8.29). This was consistent with substantial variation in mental health experiences across the population.

### Depression

Of the entire sample, 88,524 (21.31%) of 312,317 participants reported experiencing depression. Of those without a nonmelanoma skin cancer diagnosis, 61,428 (21.50%) of 285,765 reported depression. However, of those with a nonmelanoma skin cancer diagnosis, 5086 (19.15%) of 26,552 individuals reported depression. After the analysis was adjusted for the included covariates, nonmelanoma skin cancer diagnosis was not significantly associated with depression (aOR 1.01, 95% CI 0.98‐1.05; *P*<.001; Table 2).

**Table 2.** Association between nonmelanoma skin cancer and depression [8].

Characteristics	Depression			
	No, n (%)	Yes, n (%)	*P* value	aOR^a^ (95% CI)
Nonmelanoma skin cancer diagnosis			<.001	
No	224,327 (78.5)	61,438 (21.4)	<.001	Ref^b^
Yes	21,466 (80.8)	5086 (19.1)	<.001	1.01 (0.98‐1.05)
Race and ethnicity			<.001	
White only	1,97,650 (77.9)	55,984 (22)	<.001	Ref
Asian only	22,476 (83.4)	4460 (16.5)	<.001	0.50 (0.48‐0.52)
Black only	5135 (78.3)	1416 (21.6)	<.001	0.70 (0.66‐0.75)
American Indian or Alaskan Native only	7863 (88.6%)	1002 (11.3%)	<.001	0.47 (0.440.50)
Native Hawaiian or other Pacific Islander only	1721 (84.3)	320 (15.6)	<.001	0.51 (0.45‐0.58)
Multiracial	4778 (83.2)	964 (16.7)	<.001	0.60 (0.56‐0.64)
Other race only	6170 (72.1)	2378 (27.8)	<.001	1.08 (1.04‐1.15)
Sex			<.001	
Male	130,827 (84.8)	23,403 (15.1)	<.001	Ref
Female	114,966 (72.7)	43,121 (27.2)	<.001	1.98 (1.94‐2.02)
Age (years)			<.001	
18-64	150,115 (75.6)	48,279 (24.3)	<.001	Ref
≥65	95,678 (83.9)	18,245 (16)	<.001	0.44 (0.440.45)
BMI			<.001	
Underweight	3581 (74.5)	1221 (25.4)	<.001	Ref
Normal weight	72,440 (81)	16,991 (18.9)	<.001	0.83 (0.77‐0.89)
Overweight	91,376 (81.8)	20,304 (18.1)	<.001	0.87 (0.81‐0.94)
Obese	78,396 (73.6)	28,008 (26.3)	<.001	1.18 (1.10‐1.27)
Education			<.001	
Did not graduate high school	10,816 (76.2)	3368 (23.7)	<.001	Ref
Graduated high school	58,033 (79.1)	15,252 (20.8)	.27	0.98 (0.94‐1.02)
Attended college or technical school	63,782 (76.1)	19,979 (23.8)	<.001	1.02 (0.98‐1.07)
Graduated from college or technical	113,162 (80.2)	27,925 (19.7)	<.001	0.91 (0.87‐0.95)
Other cancer			<.001	1.12 (1.08‐1.15)
No	217,198 (78.7)	58,447 (21.2)	<.001	Ref
Yes	28,595 (77.9)	8077 (22)	<.001	1.12 (1.08‐1.15)
Heart disease			<.001	
No	220,365 (79.3)	57,491 (20.6)	<.001	Ref
Yes	25,428 (73.7)	9033 (26.2)	<.001	1.41 (1.37‐1.45)
Lung disease			<.001	
No	206,137 (82)	45,126 (17.9)	<.001	Ref
Yes	39,656 (64.9)	21,398 (35)	<.001	2.10 (2.05‐2.14)
Kidney disease			<.001	
No	235,503 (79.1)	62,081 (20.8)	<.001	Ref
Yes	10,290 (69.8)	4443 (30.1)	<.001	1.47 (1.41‐1.53)

a aOR: adjusted odds ratio.

b Ref: reference.

When analyzing all racial and ethnic groups, there were many considerable differences in the prevalence of depression. White respondents were used as the reference group. Using the reference group, Asian (aOR 0.50, 95% CI 0.48‐0.52), Black (aOR 0.70, 95% CI 0.66‐0.75), American Indian or Alaska Native (aOR 0.47, 95% CI 0.44‐0.50), Native Hawaiian or other Pacific Islander (aOR 0.51, 95% CI 0.45‐0.58), and multiracial respondents (aOR 0.60, 95% CI 0.56‐0.64) all had lower adjusted odds of depression. Participants in the “other” category had slightly higher odds of depression compared to White respondents (aOR 1.08, 95% CI 1.04‐1.15).

Women (43,121/158,087, 27.28%) reported significantly higher rates of depression compared to men (23,403/154,230, 15.19%). After adjustment, women had almost double the odds of depression when compared to men (aOR 1.98, 95% CI 1.94‐2.02). Participants (18,245/113,923, 16.0%) aged 65 years or older had significantly lower rates of depression compared to adults (48,279/198,394, 24.3%) aged 18 to 64 years. BMI also played a substantial role. With underweight individuals as the reference group, obese individuals experienced higher odds of depression (aOR 1.18, 95% CI 1.10‐1.27), while those who were underweight or of normal weight had lower odds compared to those who were considered overweight or obese.

After adjusting for covariates, high school graduates had similar odds of depression to the reference group (aOR 0.98, 95% CI 0.94‐1.02). Participants who had reached the college level of education had slightly different odds (aOR 1.02, 95% CI 0.98‐1.07), and college graduates had lower odds (aOR 0.91, 95% CI 0.87‐0.95).

### Poor Mental Health Days

Respondents with a history of nonmelanoma skin cancer reported a higher average number of poor mental health days (mean 4.54, SD 8.37) compared to those without a nonmelanoma skin cancer diagnosis (mean 3.20, SD 7.37). However, after adjustment, individuals with nonmelanoma skin cancer experienced a slight decrease in poor mental health days compared to those without (aRR 0.94, 95% CI 0.91‐0.97; Table 3).

**Table 3.** Association between nonmelanoma skin cancer and poor mental health days [8].

Characteristics	Mental health days		
	Mean (SD)	*P* value	aRR^a^ (95% CI)
Skin cancer diagnosis		<.001	
No	3.19 (7.36)	<.001	Ref^b^
Yes	4.54 (8.36)	<.001	0.94 (0.91‐0.97)
Race and ethnicity		<.001	
White only	4.30 (8.17)	<.001	Ref
Asian only	4.80 (8.65)	<.001	0.93 (0.90‐0.95)
Black only	5.86 (9.52)	<.001	1.07 (1.01‐1.13)
American Indian or Alaskan Native only	3.36 (6.85)	<.001	0.82 (0.78‐0.86)
Native Hawaiian or other Pacific Islander only	5.42 (9.34)	<.001	1.11 (1.00‐1.24)
Multiracial	4.65 (8.70)	<.001	0.93 (0.88‐1.00)
Other race only	6.45 (9.74)	<.001	1.25 (1.19‐1.32)
Sex		<.001	
Male	3.64 (7.71)	<.001	Ref
Female	5.18 (8.74)	<.001	1.36 (1.34‐1.39)
Age (y)		<.001	
18-64	5.43 (8.85)	<.001	Ref
≥65	2.66 (6.85)	<.001	0.40 (0.40‐0.41)
BMI		<.001	
Underweight	6.37 (9.81)	<.001	Ref
Normal weight	4.18 (7.95)	<.001	0.76 (0.71‐0.82)
Overweight	3.78 (7.70)	<.001	0.71 (0.67‐0.76)
Obese	5.20 (8.98)	<.001	0.83 (0.78‐0.89)
Education		<.001	
Did not graduate high school	6.13 (10.21)	<.001	Ref
Graduated high school	5.01 (9.06)	<.001	1.01 (1.01‐1.01)
Attended college or technical school	5.01 (8.84)	<.001	1.03 (1.03‐1.03)
Graduated from college or technical	3.59 (7.15)	<.001	0.88 (0.88‐0.88)
Other cancer diagnosis		<.001	
No	4.47 (8.28)	<.001	Ref
Yes	4.09 (8.35)	<.001	1.12 (1.08‐1.14)
Heart disease		<.001	
No	4.31 (8.11)	<.001	Ref
Yes	5.30 (9.55)	<.001	1.33 (1.30‐1.37)
Lung disease		<.001	
No	3.87 (7.73)	<.001	Ref
Yes	6.67 (9.95)	<.001	1.50 (1.47‐1.53)
Kidney disease		<.001	
No	4.36 (8.21)	<.001	Ref
Yes	5.60 (9.63)	<.001	1.28 (1.23‐1.33)

a aOR: adjusted odds ratio.

b Ref: reference.

Significant differences in mental health days were observed by race and ethnicity. Black individuals reported the highest average (5.85 d). This group had significantly increased rates of mental health issues compared to White individuals (aRR 1.07, 95% CI 1.01‐1.13). In contrast, American Indian or Alaska Native participants (aRR 0.82, 95% CI 0.78‐0.86), multiracial individuals (aRR 0.93, 95% CI 0.88‐1.00), and Asian respondents (aRR 0.93, 95% CI 0.90‐0.95) reported fewer mental health days compared to the White reference group.

Women had significantly more poor mental health days (mean 5.18, SD 8.75) compared to men (mean 3.65, SD 7.72). After adjustment, women had substantially higher rates of mental health distress (aRR 1.36, 95% CI 1.34‐1.39). Respondents aged 65 years and older reported fewer mental health days than those in lower age groups (aRR 0.40, 95% CI 0.40‐0.41).

BMI was strongly associated with mental health outcomes. Underweight individuals experienced the highest number of poor mental health days (mean 6.40, SD 9.85) and served as the reference group. Compared to them, respondents of normal weight (aRR 0.76, 95% CI 0.71‐0.82), overweight individuals (aRR 0.71, 95% CI 0.67‐0.76), and those with obesity (aRR 0.83, 95% CI 0.78‐0.89) all had significantly lower rates of poor mental health days.

Individuals who did not graduate high school reported the highest average number of poor mental health days (6.13 d), while college graduates reported the fewest number (3.60 d). After adjustment, graduating from college or technical school was associated with significantly fewer mental health days (aRR 0.88, 95% CI 0.88‐0.88) compared to individuals with less education.

Several comorbid health conditions were also associated with increased mental distress. Individuals with another cancer diagnosis had more poor mental health days (mean 5.01, SD 7.56) and higher adjusted rates compared to those without other cancers (aRR 1.12, 95% CI 1.08‐1.14). Lung disease was associated with the strongest increase in mental health burden (mean 6.77, SD 7.81; aRR 1.50, 95% CI 1.47‐1.53). Respondents with kidney disease (aRR 1.28, 95% CI 1.23‐1.33) and heart disease (aRR 1.33, 95% CI 1.30‐1.37) also reported significantly higher adjusted rates of poor mental health days compared to their respective reference groups.

## Discussion

### Principal Findings

In this nationally representative sample, study findings reveal a subtle relationship between nonmelanoma skin cancer and mental health: while individuals with a history of nonmelanoma skin cancer were slightly less likely to report a formal diagnosis of depression in unadjusted comparisons, nonmelanoma skin cancer was not significantly associated with depression after adjusting for demographics, other cancers, and chronic diseases. However, individuals with nonmelanoma skin cancer reported a higher number of poor mental health days before adjustment but slightly fewer poor mental health days after adjustment. These findings suggest that the differences in mental health burden are largely explained by sociodemographic and comorbid factors instead of the nonmelanoma skin cancer itself.

Prior research has suggested that the association between nonmelanoma skin cancer and mental health may operate in both biological and psychological directions. Chronic psychological stress has been shown to alter neuroendocrine-immune pathways, increasing inflammatory activity, impairing wound repair, and weakening immune surveillance, which may elevate susceptibility to certain nonmelanoma skin cancers [6]. However, a nonmelanoma skin cancer diagnosis may contribute to psychological distress through concerns about recurrence, uncertainty during long-term surveillance, scarring, and changes in visible appearance. These have all been documented as drivers of anxiety and depressive symptoms in melanoma and nonmelanoma patient populations [12,13].

A cancer diagnosis itself is often associated with increased stress. Prior research has shown that uncertainty about outcomes and concerns about physical appearance can elevate psychological stress, particularly in patients with visible scars [5,11]. Although stress was not directly measured in this study, the higher number of poor mental health days reported by individuals with nonmelanoma skin cancer may reflect this psychological impact. These findings support the notion that cancer-related stress can appear in daily tasks, even when it does not meet clinical criteria for depression [2,5].

Interestingly, in adjusted models, individuals with a history of nonmelanoma skin cancer reported fewer poor mental health days compared with those without nonmelanoma skin cancer, while no association was observed with depression. Several potential mechanisms may help explain this counterintuitive pattern. Nonmelanoma skin cancer is typically detected early, treated effectively, and associated with excellent long-term outcomes, which may mitigate sustained psychological distress. Successful removal of visible lesions can also create a sense of resolution or restored control, potentially improving daily emotional well-being. In addition, patients with nonmelanoma skin cancer may often engage in regular dermatologic care, providing frequent health care touchpoints that may reduce uncertainty, reinforce preventive health behaviors, and reflect a population with generally higher health literacy or wellness-oriented behaviors, factors that are linked to more favorable mental health profiles.

The sociodemographic differences observed in this study are consistent with broader public health literature, showing that mental health outcomes are shaped by structural, cultural, and economic factors. Higher rates of poor mental health days among women and younger adults may reflect increased stress, body image concerns, or work-related pressures. Racial variation may be influenced by differences in health care access. Educational and income-related disparities may also reflect gaps in early detection resources. These findings underscore the importance of tailoring mental health support within dermatologic and oncologic care to the needs of various groups rather than applying a uniform approach.

The nature of being diagnosed with nonmelanoma skin cancer itself may contribute significantly to this distress. Patients often experience fear of imperfections due to visible scarring from surgery, concerns about cancer recurrence, or anxiety over potential mortality, especially with melanoma [5,11]. The continuation of dermatological watch and uncertainty with treatments can further elevate emotional strain for individuals. This specifically takes place when the cancer affects visible areas, such as the face or neck [2]. These stressors may not meet the clinical definition of depression but can still influence day-to-day mental well-being [5].

These results align with previous studies that highlight psychological distress among patients with nonmelanoma skin cancer. However, some research has found higher rates of depression, suggesting variability based on sample demographics or methods of measurement [5,11]. This study adds to the conversation by emphasizing subjective mental distress, which may not always manifest as a clinical diagnosis, while also showing that much of the observed association may be explained by comorbid illness and sociodemographic factors.

We also observed key sociodemographic differences. Women, younger adults, individuals with higher BMI, and those with lower levels of education reported a higher number of poor mental health days and higher levels of depression. These outcomes are consistent with a large amount of public health literature and suggest that mental health improvements should be tailored to the vulnerabilities of different subgroups [3,10].

Following these results, a consistent routine of mental health screenings for those diagnosed with nonmelanoma skin cancer is recommended to help relieve mental distress. This may include screening tools such as the Patient Health Questionnaire-9 during dermatology or oncology visits. This incorporates automatic referral pathways to licensed mental health providers with outstanding scores. Integrated care models may also involve co-located behavioral health specialists (eg, psychologists, social workers, or psychiatric nurse practitioners) within dermatology or oncology clinics. Incorporating this may help address psychological needs associated with the diagnosis and its follow-up care. Moreover, support groups, cognitive behavioral therapy, or survivorship counseling should be offered as part of a thorough treatment plan, helping patients manage stressors, such as body image changes, fear, and long-term mental challenges [1,2].

This study has several limitations. As the BRFSS dataset is cross-sectional, the direction of the relationship between nonmelanoma skin cancer and mental health outcomes cannot be established. It is not possible to determine whether poor mental health causes the development of nonmelanoma skin cancer or arises because of diagnosis, treatment, and other factors of nonmelanoma skin cancer. Poor mental health days rely on self-report and capture broad, nonspecific distress, which may not align with clinical diagnoses. Reverse causality is possible if individuals with mental health issues are more likely to seek evaluation for skin changes, leading to higher rates of nonmelanoma skin cancer detection. Additionally, several confounding variables, such as family history of cancer, medication use, and factors such as sun exposure or smoking, were not used in the dataset and may partially explain the observed associations. Although this analysis adjusted for several major chronic illnesses (other cancers, lung disease, heart disease, and kidney disease), many clinically important conditions remain unmeasured. For instance, a participant may have both nonmelanoma skin cancer and a more psychologically burdensome condition, such as lung cancer or severe cardiac disease, which could influence their mental health outcomes. The inability to differentiate whether mental health symptoms stem from nonmelanoma skin cancer itself or from co-occurring illnesses limits the precision of our findings. Additionally, our analyses do not capture illness perceptions, cosmetic concerns, or treatment experiences that may influence psychological outcomes. Future work using datasets with richer clinical detail or linked cancer registry data may help more accurately isolate the independent effect of skin cancer on mental health. These findings should be interpreted as a correlation, and future research is needed to clarify the direction of this relationship. Finally, the BRFSS survey may not capture more nuanced mental health challenges such as anxiety or posttraumatic stress disorder, limiting the depth of insight into the psychological experiences of patients with nonmelanoma skin cancer [5,9]. All variables were self-reported, which may introduce misclassification of both exposures and outcomes.

It is also crucial to recognize that depression is frequently underdiagnosed in community populations, particularly among older adults, men, and individuals with limited access to health care. The BRFSS depression variable relies on self-reported clinical diagnosis, which does not capture unreported cases. More sensitive mental health assessments, such as the Patient Health Questionnaire-9 or validated cancer-specific screening tools, may better capture psychological distress in future studies.

### Conclusions

This study highlights a major association between mental health challenges, particularly

depression and poor mental health days, and the presence of nonmelanoma skin cancer among US adults using nationally representative data from the 2023 BRFSS [9]. Adults with a history of skin cancer reported higher unadjusted levels of day-to-day mental distress than those without skin cancer, but analyses adjusted for covariates showed no significant association with depression and a slight decrease in poor mental health days. Moreover, sociodemographic factors play a substantial role in shaping mental health, with certain groups showing greater vulnerability [3,10].

These results emphasize the importance of integrated care models that address both physical and mental health outcomes in patients with nonmelanoma skin cancer [1,2]. Public health initiatives should prioritize mental health screening and support within dermatologic and oncologic care, especially for disproportionately affected populations. The favorable mental health profile observed among individuals with nonmelanoma skin cancer may also highlight opportunities to leverage routine dermatologic care as a platform for promoting mental well-being and early identification of psychosocial needs. Future research should investigate longitudinal patterns, causal mechanisms, and the effectiveness of mental health interventions in improving quality of life and potentially clinical outcomes among patients with nonmelanoma skin cancer, and whether resilience, health care engagement, or other unmeasured attributes mediate these associations, and whether similar patterns emerge across diverse populations and cancer types [5,11].

Ultimately, recognizing and addressing the mental health burden associated with nonmelanoma skin cancer can lead to more holistic, equitable, and patient-centered care strategies.

## Abbreviations

aOR: adjusted odds ratio
aRR: adjusted rate ratio
BRFSS: Behavioral Risk Factor Surveillance System
